# The assessment of side effects of tyrosine kinase inhibitors (TKI) applied in patients with advanced thyroid cancer (TC) - one centre experience

**DOI:** 10.1186/1756-6614-6-S2-A33

**Published:** 2013-04-05

**Authors:** Jolanta Krajewska, Aleksandra Kukulska, Ewa Paliczka-Cieślik, Daria Handkiewicz-Junak, Tomasz Gawlik, Tomasz Olczyk, Aleksandra Kropińska, Aleksander Skoczylas, Barbara Michalik, Barbara Jarząb

**Affiliations:** 1 M. Sklodowska-Curie Memorial Cancer Centre and Institute of Oncology, Gliwice Branch, Gliwice, Poland

## 

TKI constitute a new group of drugs evaluated in TC patients. The efficacy of some of them in prolongation of progression free survival has been recently documented. However, possible side effects may affect the quality of life as well as limit their clinical use. Only drugs which were known to inhibit VEGFR were considered. In the study adverse effects were evaluated in patients treated in our centre within the prospective clinical trials phase II and III. The aim of the study was to analyze the frequency and severity of side effects related to TKI in TC patients. Thus, we retrospectively re-evaluated side effects on the basis of Common Terminology Criteria for Adverse Events (CTCAE) version 3.0. The comparison of the drugs was not aimed.

37 therapies with TKI due to advanced TC were assessed. 19 subjects were given vandetanib, 15 - lenvatinib, 3 - axitinib. Median treatment duration was 26.7 months (range: 4.0 - 62.2). The drug was discontinued due to TC progression in 9 subjects, adverse events in 5 and for other reasons in 4. Adverse events leading to treatment withdrawal were: weight loss (1), lymphopenia (1), QTC prolongation (1), tracheo-esophageal fistula (1) and purulent meningitis (1).

The results are given in table 1.

**Table 1:** Frequency and severity of the most common treatment-related side effects.

	G1 / G2 / G3	Total	Treatable	Dose reduction
Skin reactions	16 / 8 / 2	26 (70.3%)	yes	no
Arterial hypertension	3 / 7 / 17	27 (73.0%)	yes	1
Diarrhea	7 / 9 / 4	20 (54.1%)	yes	4
Weight loss	4 / 13 / 3	20 (54.1%)	yes	5
Stomatitis	15 /1 / -	16 (43.2%)	yes	no

## Conclusions

Tolerability and safety of the treatment with tyrosine kinase inhibitors in advanced TC patients was acceptable (most adverse reactions G1-G2). Only in 13.5% cases treatment discontinuation was required.

